# Enhanced Spatial and Extended Temporal Graph Convolutional Network for Skeleton-Based Action Recognition

**DOI:** 10.3390/s20185260

**Published:** 2020-09-15

**Authors:** Fanjia Li, Juanjuan Li, Aichun Zhu, Yonggang Xu, Hongsheng Yin, Gang Hua

**Affiliations:** 1 School of Information and Control Engineering, China University of Mining and Technology, Xuzhou 221008, China; lifanjia@163.com (F.L.); lijj@cumt.edu.cn (J.L.); xygang@cumt.edu.cn (Y.X.); yhs@cumt.edu.cn (H.Y.); 2 Jiangsu Province Xuzhou Technician Institute, Xuzhou 221151, China; 3 School of Computer Science and Technology, Nanjing Tech University, Nanjing 211800, China; aichun.zhu@njtech.edu.cn

**Keywords:** skeleton-based action recognition, graph convolution network, enhanced spatial, extended temporal

## Abstract

In the skeleton-based human action recognition domain, the spatial-temporal graph convolution networks (ST-GCNs) have made great progress recently. However, they use only one fixed temporal convolution kernel, which is not enough to extract the temporal cues comprehensively. Moreover, simply connecting the spatial graph convolution layer (GCL) and the temporal GCL in series is not the optimal solution. To this end, we propose a novel enhanced spatial and extended temporal graph convolutional network (EE-GCN) in this paper. Three convolution kernels with different sizes are chosen to extract the discriminative temporal features from shorter to longer terms. The corresponding GCLs are then concatenated by a powerful yet efficient one-shot aggregation (OSA) + effective squeeze-excitation (eSE) structure. The OSA module aggregates the features from each layer once to the output, and the eSE module explores the interdependency between the channels of the output. Besides, we propose a new connection paradigm to enhance the spatial features, which expand the serial connection to a combination of serial and parallel connections by adding a spatial GCL in parallel with the temporal GCLs. The proposed method is evaluated on three large scale datasets, and the experimental results show that the performance of our method exceeds previous state-of-the-art methods.

## 1. Introduction

Human action recognition has many application scenarios in the real world, such as security surveillance, health care systems, autonomous driving and human-computer interaction [1,2,3,4,5]. There are two main research directions in this field: using RGB video or skeleton sequences as model inputs. In recent years, skeleton-based action recognition is attracting more and more interest due to the following three advantages [6,7,8,9,10,11]: Firstly, the skeleton data can be easily acquired by low-cost depth sensors [12] (e.g., Microsoft Kinect, Asus Xtion, etc.) or pose estimation algorithms [13,14,15]. Secondly, biological studies have proved that the skeleton, as a high-level representation of the human body, is informative and discriminative to express human activities [16]. Thirdly, compared with the RGB videos, the skeleton data are more robust in terms of illumination, clothing textures, background clutter, variations in viewpoints, etc. [17,18,19]. For the above reasons, our work focuses on the task of skeleton-based action recognition. 

The earlier deep-learning-based methods in this field use Recurrent Neural Networks (RNN) or Convolutional Neural Networks (CNN), which have achieved much better performance than hand-crafted methods [20,21,22]. Nevertheless, whether they model the skeleton data as a sequence of vectors like the RNNs do, or model them as 2D pseudo images like the CNNs do, they all neglect that the human skeleton is naturally a non-Euclidean graph-structured composed of vertices and edges. Based on this judgment, Yan et al. [23] proposed a spatial-temporal graph convolutional network (ST-GCN) representing human joints as vertices and the bones as edges. The ST-GCN improves the accuracy of action recognition to a new level, and substantial ST-GCNs are subsequently proposed based on it [24,25,26,27,28,29,30,31,32,33,34,35]. However, there are still two problems to be addressed in these methods.

The first problem lies in the temporal domain. The forms of human actions are many and varied. According to the human cognitive process of action understanding, some actions can be recognized by just a glimpse, such as “writing”. While some actions, such as “wear a shoe”, need a much longer observation before they can be recognized. For another example, the action “nod head/bow” is a sub-action of the action “pickup”, which requires the model to be sensitive to both short-duration and long-duration actions. However, the temporal GCL of the ST-GCNs utilizes only one fixed kernel size 9 × 1 which is obviously not enough to cover all the temporal dynamics. Besides, the kernel size is relatively large, which increases the number of parameters and reduces model efficiency.

The second problem lies in the spatial domain. Tran et al. [36] demonstrate that by means of 3D CNNs, the RGB-based action recognition can yield accuracy advantages over 2D CNNs. Moreover, by factorizing the 3D CNNs to spatial-only and temporal-only components, further performance improvements can be achieved. The ST-GCNs borrow this finding and construct the basic block of the networks by serially connecting a spatial GCL and a temporal GCL. Although this kind of construction is concise and efficient, it mixes the spatial cues into temporal cues, resulting in weakened spatial representation and limited model performance. For example, the actions ”touch head” and ”touch neck” look very similar. Their slight difference lies in the distance between the hand and the head or neck, which requires the model to be sensitive to the spatial aspect. However, the ST-GCNs are confused when distinguishing them.

**Figure 1:**
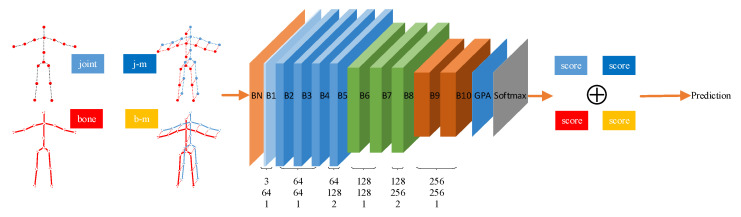
The architecture of the enhanced spatial and extended temporal graph convolutional network. There are a total of 10 basic blocks in the network, which are marked as B1 to B10. Take B8 as an example to introduce the meaning of the three numbers (i.e., 128, 256, 2) below each block. The number of input channels and output channels of B8 is set to 128 and 256, and the temporal stride is set to 2. There are four modalities fed into the network separately: joint, bone, joint motion (j-m), and bone motion (b-m). The corresponding four softmax scores are added to get the final prediction results.

**Figure 2:**
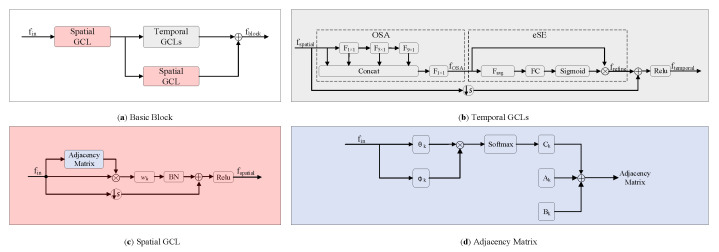
The details of the basic block for EEGCN ⊕ indicates the elementwise addition operation and ⊗ indicates the matrix multiplication operation. In Figure 2b, F_1×1_, F_5×1_ and F_9×1_ denote 1 × 1, 5 × 1 and 9 × 1 convolution layer respectively, F_avg_ is global average pooling, FC is fully-connected layer. In Figure 2d, A_k_ denotes the fixed natural structural graph illustrated in Figure 3, B_k_ denotes the layer adaptive adjacency matrix, C_k_ denotes the sample adaptive adjacency matrix, θ_k_ and φ_k_ denote the two embedding functions.

To address the above two problems, we propose a novel model namely enhanced spatial and extended temporal graph convolutional network (EE-GCN). Figure 1 shows the overall framework and Figure 2 shows the details of the basic block of the model. For the first problem, we employ multiple convolution kernels with different sizes instead of the fixed one to comprehensively aggregate discriminative temporal cues from shorter to longer terms. Notably, smaller kernel sizes (no bigger than 9 × 1) are chosen for fewer parameters and higher model efficiency. As one kernel corresponds to one convolution layer, how to combine these layers is another problem we face. Inspired by the success of VoVNetV2 [37], we propose to concatenate the consecutive layers by a one-shot aggregation (OSA) + effective squeeze-excitation (eSE) structure which well balances the performance and efficiency. As shown in Figure 2b, the OSA module connects the layers by two pathways. The first pathway is to connect these layers in series to obtain a larger receptive field. The second pathway is the connection between each layer and the final output, so the final output features are the aggregation of all the temporal features extracted by different convolution kernels. The eSE module is then used to explore the interdependency between the output channels, which can be seen as a channel attention mechanism. For the second problem, we propose a new paradigm of the basic block structure which incorporates a parallel structure to the original tandem structure. Specifically, after the spatial GCL, we insert an additional spatial GCL in parallel with the temporal GCLs. With the combination of tandem and parallel relationships between the spatial and temporal GCLs, our model is much more effective for capturing complex spatial-temporal features.

To verify the superiority of our EE-GCN, extensive experiments are conducted on three large-scale benchmark datasets for skeleton-based recognition: NTU-RGB+D 60 [9], NTU-RGB+D 120 [38], and Kinetics-Skeleton [39]. Our method proves its merits from the results. 

Overall, the main contributions of this work are summarized as follows:

(1) We propose an EE-GCN which can substantially facilitate spatial-temporal feature learning. To efficiently and comprehensively extract the temporal discriminative features, we propose to construct the temporal graph convolution with multiple smaller kernel sizes instead of the typically larger one. Moreover, the corresponding temporal GCLs are concatenated by an OSA + eSE structure which can well balance performance in terms of accuracy and speed.
(2) On the basis of the extended temporal convolution, we propose a new paradigm of the basic block structure to enhance the spatial convolution. While retaining the original spatial GCL, we add the same layer in parallel with the temporal GCLs. This hybrid structure, which contains both tandem and parallel connections, can further increase model performance.
(3) We empirically demonstrate that our model outperforms previous state-of-the-art methods.

## 2. Related Work

In this section, we review methods that are dedicated to the skeleton-based action recognition task in detail. Limited by the shallow architecture and lack of universality, the hand-craft methods [20,21,22] in this domain are substituted by deep learning-based methods in recent years. We divide these deep learning-based methods into two categories: non-GCN-based and GCN-based. According to our main contributions, we provide an overview of the GCN-based methods from two aspects: spatial domain and temporal domain.

### 2.1. Non-GCN-Based Methods

The earlier deep learning-based methods in this field are mainly constructed by RNN and CNN. The RNN, represented by Long Short-Term Memory (LSTM) [40] or Gated Recurrent Unit (GRU) [41], has an advantage in modeling temporal dynamics of skeleton data [8,18,19,42,43,44,45,46,47]. A view adaptive network based on LSTM is proposed in [45], which can automatically choose the optimal angle to reduce the impact of diverse angles. Ref. [19] proposes a Spatio-temporal LSTM (ST-LSTM) network which can better extract the long-term contextual information in the temporal domain, and capture dependencies of joints more deeply in the spatial domain. Ref. [46] employs an attention enhanced graph convolution to boost the performance of LSTM. However, affected by the gradient exploding and vanishing problems, the RNN is difficult to train and its usage is thus limited. Compared with the RNN, the CNN is easier to train and parallelize. It structures the skeleton sequences as the R, G, B channels of pseudo-images [11,48,49,50,51,52]. For example, Ke et al. [11] propose a new representation of the skeleton sequences by transforming them into three clips corresponding to the three channels of the sequence 3D coordinates. Ref. [48] is the first application of 3D CNN in the task, which can simultaneously extract spatial and temporal features. To learn both local and global co-occurrence features, Li et al. [51] propose a network which first encodes point-level information, and then aggregates global-level information through a transpose operation. The performance of this network is the best before the proposal of ST-GCN. However, both the RNN and CNN neglect the graphic structure of the human skeleton, resulting in no further performance improvement.

### 2.2. Improvements of GCN-Based Methods in the Spatial Domain

The spatial graph construction of ST-GCN [23] is according to the natural physical structure of the human skeleton, which is proved effective. But as the adjacency matrix is pre-defined based on prior knowledge, the dependencies of disconnected joint pairs which may be crucial for action recognition are not well exploited. Subsequently, substantial methods are proposed to overcome this drawback. Wen et al. [27] propose a motif-based GCN in which the adjacency matrix is defined according to the Euclidean distance between joint pairs. Therefore, both of the connected and disconnected joints are modeled simultaneously. Ref. [53] proposes a part-based graph convolutional network with the whole skeleton graph divided into four subgraphs corresponding to four parts of the entire human body. The network infers relations between the subgraphs and captures the high-level features.

On the basis of retaining the fixed natural structural graph, Shi et al. [24] add another two novel graphs that are adaptive to each layer and each action respectively. The fusion of these three graphs can better suit the action recognition task. Ref. [32] proposes a multi-scale GCN using two scales of graphs to extract discriminative information from skeleton data: a joint-scale graph to extract joint-level features, and a part-scale graph to extract part-level features. Furthermore, a bidirectional fusion mechanism is introduced to merge these features. Note that the graphs incorporated by the above methods are all manually set and fixed through the entire networks. Ref. [33] changes this kind of operation and proposes an automatically designed GCN by neural architecture searching. Multiple dynamic graph modules are provided to the search space and the optimal one is chosen for each layer. Ref. [54] completely frees from the shackles of constructing graphs based on the human skeleton structure. A joint-level module is proposed to learn the adjacent matrix by incorporating the semantics of joint type which is demonstrated beneficial for learning graph edges (i.e., connecting weights of joint pairs). However, all these methods only adopt tandem structure to connect the spatial convolution and the temporal convolution, which results in limited performance.

### 2.3. Improvements of GCN-Based Methods in the Temporal Domain

The temporal graph convolution of ST-GCN [23] only utilizes a fixed 9 × 1 kernel which is not optimal in modeling the diverse temporal dynamics. To this end, Liu et al. [29] propose a multi-scale temporal modeling scheme. The kernel size in this scheme is still fixed, but multiple dilation rates are employed to achieve larger receptive fields. Cheng et al. [30] propose an adaptive temporal shift graph convolution which can adjust the receptive field adaptively according to different model layers and different datasets. The operation is more powerful and more efficient compared with the regular temporal convolution. Shi et al. [28] argue that in the temporal dimension, different frames exert different levels of importance for the final recognition. So they propose a simple yet effective STC-attention module which can prompt the model to pay more attention to important frames. The module is placed between spatial convolution and temporal convolution in each basic block. Another critical drawback of ST-GCN is that the model limits the optimization of the spatial graph to the intra-frame and disregards the latent temporal graph on the inter-frame. To solve this limitation, [31] proposes a temporal extension module which not only adds edges for the temporal dimension between the same joints but also between adjacent joints. The module can be easily attached between spatial convolution and temporal convolution without changing their structure. Although the above methods can capture high-level semantic information through deep structures, Zhang et al. [54] demonstrate that it is better to explicitly incorporate the semantics of temporal frame index into the network. They propose a semantics-guided frame-level module to exploit the information of sequence order, thereby improving the network’s capabilities. However, all these methods essentially only utilize one kernel for the temporal convolution, resulting in the insufficient capacity to cover all the discriminative stages of the various actions.

## 3. Methods

In this section, we introduce the pipeline and components of our proposed EE-GCN in detail.

### 3.1. Network Architecture

Figure 1 illustrates the architecture of EE-GCN. Following the setting of [23], the input data of size C_in_ × T × V (C_in_ equals to 3 in this work) is firstly normalized by a batch normalization (BN) layer. Here C_in_ denotes the number of channels with a typical value of 3 in this work, T denotes the number of frames in each sequence, and V denotes the number of joints in the human body. It is then fed into 10 serial connected basic blocks whose details are shown in Figure 2. Each block consists of two components: spatial GCL and temporal GCLs. The number of output channels for these blocks is 64, 64, 64, 64, 128, 128, 128, 256, 256, 256, respectively. Specially, the convolution stride of the 5-th and 8-th blocks is set to 2 to reduce the sequence length. After the last block, a global pooling average (GPA) layers is employed to pool the features maps to the same size. Then the prediction result is obtained with a softmax layer.

Inspired by the four-stream-based methods in [28,30], we use the same multi-stream strategy in which different data modalities are used to train separate models with the same architecture as shown in Figure 1. These four data modalities are joints, bones, joint motions, and bone motions. Specifically, the joint data are the original skeleton coordinates provided by the datasets, which is represented as:

(1) $$J_{i,t}=\left(x_{i,t},y_{i,t},z_{i,t}\right),\forall i\in V,t\in T$$

**Figure 3:**
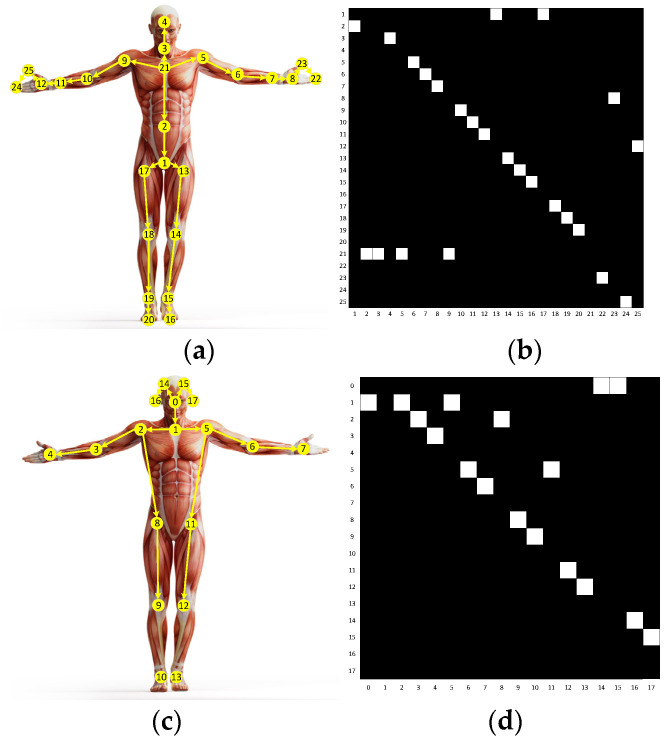
Images (**a**,**c**) represent the human skeletons in the NTU-RGB-D 60 and Kinetics-Skeleton datasets respectively. Images (**b**,**d**) represent the adjacency matrices corresponding to Images (**a**,**c**).

We use yellow circles with numbers to indicate the joints in Figure 3a,c. The bones are calculated from the differences between two adjacent joints, and their directions are away from the center of gravity of the skeleton. The yellow arrows in Figure 3a,c illustrate the bones. Given the source joint *J_i,t_* and the target joint *J_j,t_* = (*x_j,t_*, *y_j,t_*, *z_j,t_*), the bone data can be calculated as:

(2) $$B_{i,j,t}=\left(x_{j,t}-x_{i,t},y_{j,t}-x_{i,t},z_{j,t}-z_{i,t}\right)$$

Moreover, joint motions which can provide kinematic cues are calculated by the joint coordinate differences between two adjacent frames:

(3) $$J\text{-}M_{i,t}=J_{i,t+1}-J_{i,t},\forall i\in V,t\in T$$

The bone motions are also obtained in the same way:

(4) $$B\text{-}M_{i,t}=B_{i,t+1}-B_{i,t},\forall i\in V,t\in T$$

The final result is the sum of all the four softmax scores corresponding to the four modalities.

### 3.2. Spatial Graph Convolution Layer

Inspired by the success of adaptive graph [24], we formulate the spatial graph convolution which is illustrated in Figure 2c as:

(5) $$f_{Convs}=\sum_{k}^{K_{v}}W_{k}f_{in}(A_{k}+B_{k}+C_{k})$$

where *f_in_* is the input feature map, *W_k_* is the weight vector of the convolution operation, *K_v_* is the kernel size of the spatial dimension, which is set to 3 according to the joint partition strategy (i.e., root, centripetal, and centrifugal joints). *A_k_* denotes the fixed natural structural graph of the human skeleton, which is illustrated in Figure 3. *B_k_* is an adaptive graph for global attention with all the values parameterized. *C_k_* is a sample adaptive graph that measures the similarity/affinity of two joints in the embedded space. The fusion of these three graphs makes the spatial graph convolution achieve powerful performance. Finally, the spatial GCL can be formulated as:

(6) $$f_{spatial}=ReLU\left[BN\left(f_{Convs}\right)+I(f_{in})\right]$$

where *BN* denotes batch normalization (BN), *I* denotes the identity mapping for the model stability, *ReLU* denotes the rectified linear unit activation function.

### 3.3. Temporal Extension Strategy

In the traditional ST-GCNs, the temporal GCL is immediately after the spatial GCL, which performs *Γ* × 1 convolution to *f_spatial_* in Equation (6). Therefore, the sampling range for the temporal dimension can be represented as:

(7) $$R\left(v_{t}\right)=\left\{v_{q}|\left|q-t\right|\leq \frac{\Gamma }{2}\right\}$$

where *v_t_* denotes the joints in frame *t*. Then the temporal graph convolution can be formulated as:

(8) $$f_{Convt}\left(v_{t}\right)=\sum_{v_{q}\in R\left(v_{t}\right)}f_{spatial}\left(v_{q}\right)*w\left(v_{q}\right)$$

As introduced in Section 1, human actions exhibit different characteristics in the temporal domain. Some action classifications require only a few frames, while others may rely on the overall action evolution process. Therefore, just utilizing the fixed kernel size *Γ* × 1 is not enough to cover all the temporal dynamics. In addition, although the typical value of *Γ* (i.e., 9) has been proven effective, its large size brings more parameters. To address these issues, we propose to employ multiple kernels with different smaller sizes to model the temporal dimension. As one kernel corresponds to one temporal convolution layer, we are committed to organizing these layers. Inspired by the proposal of VoVNetV2 [37] in the field of instance segmentation, we propose to organize these layers by a one-shot aggregation (OSA) + effective squeeze-excitation (eSE) structure with stronger performance and higher efficiency. Below we introduce this structure which is shown in Figure 2b in detail.

#### 3.3.1. OSA Module

Suppose there are L continuous temporal GCLs with different kernel sizes, each layer follows Equation (8) and is connected by two kinds of pathways. One pathway is the connection to the subsequent layer, which leads to a larger receptive field. The other pathway is the connection to the final output, which means the features are aggregated only once into the final output features. The final output can be formulated as:

(9) $$f_{OSA}=H\left[C\left(f_{spatial},f_{1},f_{2},\ldots ,f_{L}\right)\right]$$

where *f_1_*, *f_2_*, …, *f_L_* are feature output from each layer, *C* is the concatenation operation, *H* is a composite function consisting of a 1 × 1 convolution, a BN, a ReLU. The utilization of the OSA module enables our model to comprehensively aggregate the temporal information from shorter to longer terms.

#### 3.3.2. eSE Module

The eSE module follows the OSA module, which is used to boost the model performance further. In essence, it is a channel attention mechanism that can explicitly explore the interdependency between the channels of *f_OSA_*. Firstly, the module squeezes the temporal and spatial dependencies of *f_OSA_* ∈ ℝ^*C*×*T*×*V*^ by global average pooling. In this way, the information of all frames in each sample and all joints in each frame are aggregated and the dimension of the sequence becomes [C, 1, 1]. Then one fully-connected layer followed by a sigmoid function is employed to create a channel attentive feature descriptor that can highlight the important channels. Finally, the descriptor and *f_OSA_* are element-wise multiplied to obtain the attention guided feature map with enhanced representation. The eSE module is formulated as:

(10) $$\begin{array}{l} {ℱ}_{gap}(f)=\frac{1}{TV}\sum_{i,j=1}^{T,V}f_{i,j}\\ f_{refine}=\sigma \left\{W_{C}\left[{ℱ}_{gap}\left(f_{OSA}\right)\right]\right\}⊗f_{OSA} \end{array}$$

where *ℱ_gap_* denotes the channel-wise global average pooling, *W_C_* ∈ ℝ^*C*×1×1^ denotes weights of the fully-connected layer, σ denotes the sigmoid function.

Besides, as shown in Figure 2b, the input feature map is element-wise added to the refined feature map *f_refine_* by employing a residual connection to the temporal GCLs. Therefore, the output of the temporal dimension can be formulated as:

(11) $$f_{temporal}=ReLU\left[f_{refine}+I(f_{spatial})\right]$$

### 3.4. Spatial Enhancement Strategy

**Figure 4:**
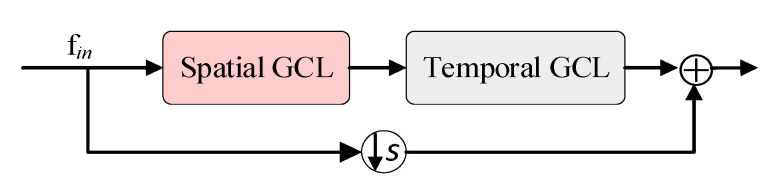
The structure of the traditional basic block.

As shown in Figure 4, the structure of the traditional block is a serial connection composed of a spatial GCL and a temporal GCL. In contrast, as shown in Figure 2a, our proposed new paradigm of the block structure includes two spatial GCLs, one in series with the temporal GCLs, and the other in parallel with the temporal GCLs. The motivation for this design lies in two aspects. On one hand, the traditional block mixed the spatial information to the temporal information through the simple serial superposition, which limits the representation ability of the spatial dimension. On the other hand, as introduced in Section 3.3, the utilization of OSA + eSE module greatly enhances the temporal information, making the spatial information relatively weak. To this end, we propose to introduce an additional spatial GCL and a parallel connection to the block. Since our spatial GCL and temporal GCLs both contain shortcuts, our proposed block does not contain shortcuts like the traditional block. The output of our block can be formulated as:

(12) $$f_{block}=f_{temporal}+S\left(f_{spatial}\right)$$

where *S* denotes the spatial graph convolution process which follows the Equation (6). The first addend *f_temporal_* denotes the extended temporal information, the second addend *S(f_spatial_)* denotes the enhanced spatial information, and the addition denotes the parallel connection. The effectiveness of our proposed new paradigm of the block structure will be demonstrated in Section 4.4.

## 4. Experiments

In this section, we first evaluate the performance and efficiency of our proposed model by exhaustive ablation studies. Unless otherwise stated, the ablation studies are performed on the X-Sub setting of NTU-RGB + D 60 using only the joint data. We then make a head-to-head comparison with other state-of-the-art methods on three large-scale datasets: NTU-RGB + D 60 [9], NTU-RGB + D 120 [38], and Kinetics-Skeleton [39].

### 4.1. Datasets

#### 4.1.1. NTU-RGB + D 60

NTU-RGB + D 60 [9] is the most widely used dataset for skeleton-based human action recognition. It contains 56,800 skeleton sequences which are categorized into 60 action classes. The samples are performed by 40 different subjects in a lab environment and captured by three cameras with different view angles. There are 25 joints with 3D coordinates in each human skeleton, and one or two subjects in each sample. The authors of the dataset recommend reporting the model performance under two settings: (1) cross-view (X-View), the samples captured by cameras 2 and 3 are used for training, and those captured by camera 1 are used for testing. (2) cross-subject (X-Sub), the samples captured from half of the subjects are used for training, and the remaining samples are used for testing. 

#### 4.1.2. NTU-RGB + D 120

NTU-RGB + D 120 [38] is currently the largest in-door-captured dataset for the task, which is an extension of NTU-RGB + D 60. It contains 114,480 skeleton samples which are categorized into 120 action classes. These samples are performed by 106 subjects and captured from 32 different camera setups. The authors of the dataset recommend reporting the model performance under two settings: (1) cross-setup (X-Setup), the samples captured from the camera setups with even IDs are used for training, and the rest are used for testing. (2) cross-subject (X-Sub), the samples performed by 53 subjects are used for training, and the remaining samples are used for testing.

There are no more than two persons in each sample of the two datasets above. We follow the pre-processing method of [24] for the datasets: if there is only one body in the sample, we add an extra body which is padded with 0.

#### 4.1.3. Kinetics-Skeleton

Kinetics [39] is a more challenging human action recognition dataset than NTU-RGB + D 60 and 120. A total of 300,000 video samples of this dataset are collected from YouTube and categorized into 400 action classes. The skeleton data are extracted using the OpenPose [13] toolbox, which contains 18 joints per human body. Each joint is represented by 2D spatial coordinates and a confidence score. There are 240,000 samples for training and the rest for testing. We set the size of the input tensor of Kinetics the same as [23], which contains two bodies in each frame.

### 4.2. Training Details

All experiments are conducted on PyTorch 1.0 platform with 4 GeForce RTX1080Ti GPUs. We use stochastic gradient descent (SGD) with Nesterov momentum (0.9) as the optimization strategies of our method. For every two steps, we update the network parameters and reset the gradients. The cross-entropy is used as the loss function and the weight decay is set to 0.0001.For NTU-RGB + D 60, 120, and Kinetics-Skeleton, we set the batch size to 36, 36, and 108, the initial learning rate to 0.3 which is divided by 10 at epochs {30, 45, 55}, {30, 50, 65}, {45, 60, 70}, and the total epochs to 70, 75, 80, respectively.

**Figure 5:**
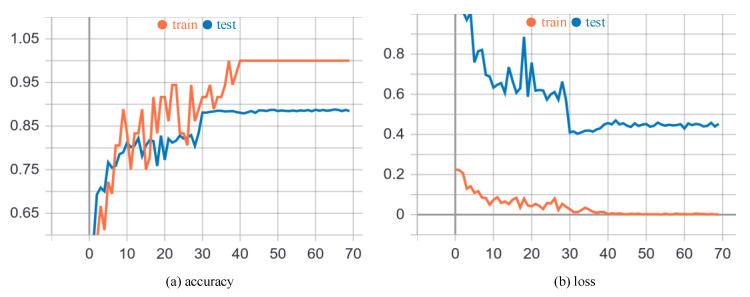
The performance and convergence speed of our EEGCN on the X-Sub setting in training process. The x-axes of (**a**,**b**) are the numbers of epochs. The y-axes of (**a**,**b**) are the recognition accuracy and cross-entropy loss respectively.

Figure 5 shows the performance and convergence speed of our model in the training and testing process. It is evident that our model converges very quickly. Specifically, as shown in Figure 5a, the learning rate is divided by 10 at epoch 30, which brings about a sharp increase of accuracy and converges the model to a relatively stable status. Although the accuracy of the training process rises to approximately 100% at epoch 40, the testing process keeps the accuracy around 89% which is the limit of our model. Figure 5b also demonstrates the same trend. There is a sudden drop of the cross-entropy loss at epoch 30, and the loss of the training process decreases to approximately 0 at epoch 40. The testing process keeps the loss around 0.44 since then. 

### 4.3. Ablation Study

We employ the following four networks to demonstrate the effectiveness of our proposed method:

Adaptive GCN (AGCN) [24]. We use this method as the baseline because our model adopts its adaptive graph construction which is introduced in Section 3.2. 

**Figure 6:**
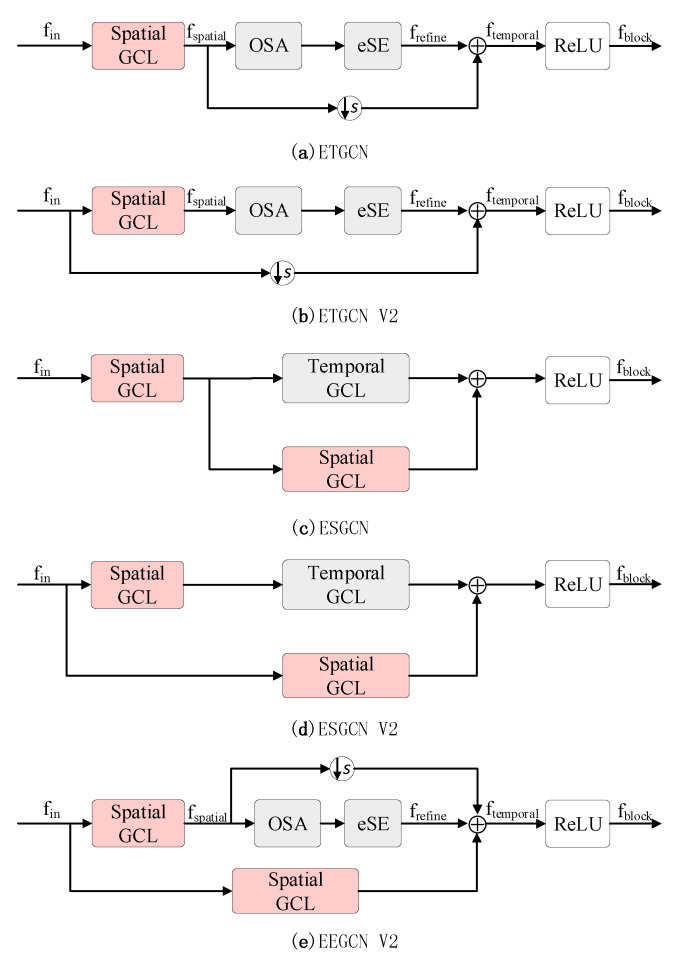
Various variants of our proposed method.

Extended Temporal GCN (ETGCN), which is shown in Figure 6a. Comparing with AGCN, the only difference lies in the utilization of our proposed temporal extension strategy which is introduced in Section 3.3.

Enhanced Spatial GCN (ESGCN), which is shown in Figure 6c. Comparing with AGCN, the only difference lies in the utilization of our proposed spatial enhancement strategy which is introduced in Section 3.4.

EEGCN, which is shown in Figure 2. This is our proposed network which integrates both extended temporal and enhanced spatial modeling strategies. 

#### 4.3.1. Temporal Extension Strategy

In this section, we use ETGCN to evaluate the performance of our temporal extension strategy. One of the key points of the strategy is the performance of using multiple temporal convolution kernels with different sizes. As the original kernel size is 9 × 1 which has been widely demonstrated to be powerful, we keep it in our proposed methods. For the selection of other kernel sizes, since more kernels and larger kernel sizes all bring more parameters, we employ no more than two kernels and no larger than 5 in kernel sizes to keep the efficiency of our model. Besides, we empirically set the number of output channels for each temporal GCL to be equal, which is 1/2 of the number of output channels for the corresponding block. The action recognition accuracies by ETGCN are reported in Table 1. We can see that using only one kernel (row 2) achieves the worst performance (86.27%). Using two or three kernels brings significant improvements, ranging from 2.07% (row 3) to 2.41% (row 7). Overall, using three kernels is generally better than using two kernels. The effectiveness of the first key point is thus verified. 

The other key point is the efficiency of the strategy. Obviously, the method using the original kernel size has the largest number of parameters (3.47 M). Benefiting from the use of OSA + eSE structure and smaller kernel sizes, the ETGCN reduces the number of parameters, ranging from 0.09 M (row 8) to 0.81 M (row 3), while improving performance. The utilization of kernel size 1 × 1 (rows 3, 6, 7) can also be seen as a bottleneck that reduces the computational costs. The second key point is thus verified. Considering that the combination of 1 × 1, 5 × 1, and 9 × 1 (row 7) achieves the best performance (88.68%) while reducing the number of parameters by 0.35 M, we adopt this combination.

Besides, to further investigate our temporal extension strategy, we propose another version of ETGCN named ETGCN V2 which is illustrated in Figure 6b. Our motivation for this version is to follow the traditional block structure shown in Figure 4, which includes a shortcut. Specifically, based on ETGCN, we first remove the shortcut of the temporal GCLs and then add a shortcut to the block. As shown in row 4 of Table 2, the performance of ETGCN V2 is 87.47%, which is 1.2% higher than that of AGCN, proving the effectiveness of OSA + eSE. But it is 1.21% lower than ETGCN, which proves the superiority of the latter.

**Figure 7:**
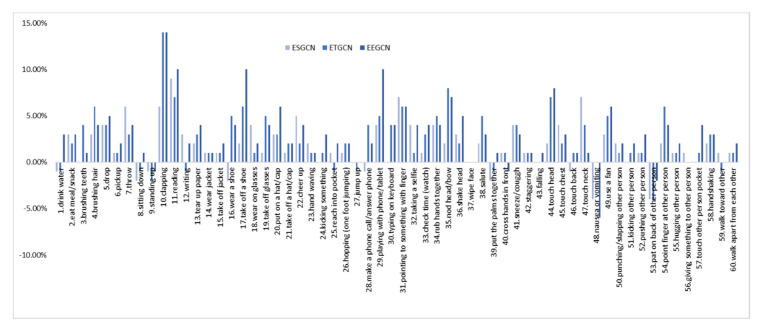
The improvements of each action in ESGCN, ETGCN, and EEGCN over AGCN [24].

**Figure 8:**
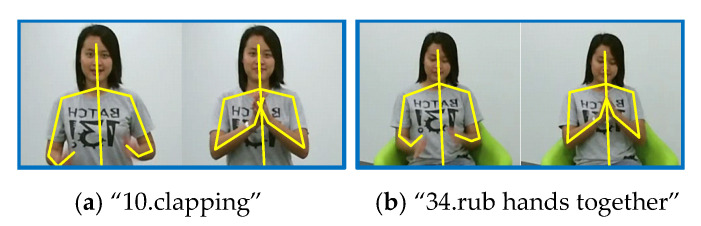
Two examples for class “10.clapping” and class “34.rub hands together”. The yellow lines represent the skeletons.

**Figure 9:**
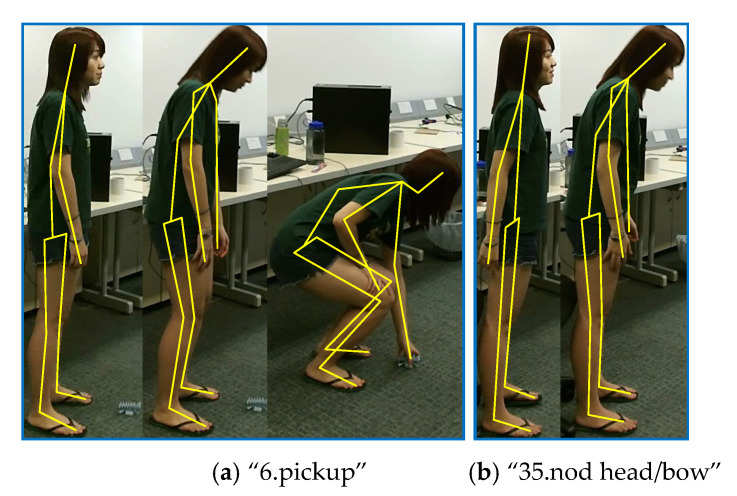
Two examples for class “6.pickup” and class “35.nod head/bow”.

Furthermore, we make an in-depth analysis of our ETGCN based on each action. Figure 7 and Table 3 list the improvements of ETGCN over AGCN. We can observe that in all the 60 actions, there are 47 actions that achieve improvements by 14% to 1%, 4 actions have no changes, and only 9 actions get slightly lower accuracies. Take the action “10. clapping” for example. As shown in Table 4, our ETGCN achieves 81% accuracy with 14% improvements over AGCN [24]. In AGCN [24], 16% of “10.clapping” samples are misclassified as “34.rub hands together”, while in ETGCN, this percentage is reduced to 7%. Figure 8 illustrates these two actions. They look very similar in the initial execution phase, which all bring the open hands together. But from the perspective of temporal evolution, they are very different. The action “10.clapping” is to alternately separate and close two hands with a characteristic frequency pattern. But the action “34.rub hands together” is to move the two hands back and forth while keeping them closed. Thanks to the advantages of the long-term motion trend modeling, our ETGCN can classify them more accurately. We give another example to illustrate further. As shown in Table 4, 88% of “35.nod head/bow” samples are correctly classified by AGCN [24], while in ETGCN, the percentage is 96%. This action is easily confused with five classes of actions: “6.pickup”, “16.wear a shoe”, “17.take off a shoe”, “43.falling”, and “48.nausea or vomiting”. Obviously, these actions are similar at the beginning of the executions, which all start with head bowing and bending. In other words, the action “35.nod head/bow” is a sub-action of the other five classes of actions. As shown in Figure 9, the follow-up motions of “6.pickup” include leg bending and hand stretching, while the action “35.nod head/bow” does not include these motions. Therefore, the long-term motion trend modeling ability of ETGCN is useful in this situation and Table 4 proves its effectiveness.

#### 4.3.2. Spatial Enhancement Strategy

**Figure 10:**
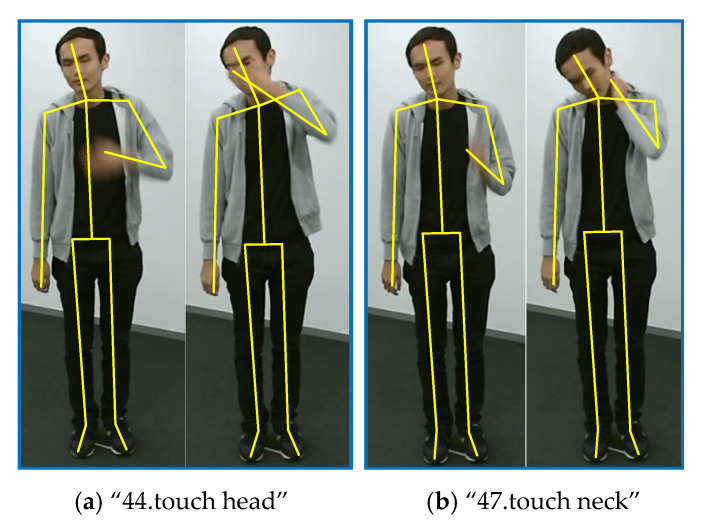
Two examples for class “44.touch head” and class “47.touch neck”.

As introduced in Section 3.4, we enhance the spatial features by adding an additional spatial GCL in parallel with the temporal GCLs for each basic block. Row 5 of Table 2 demonstrates the superiority of this new block structure paradigm. Our ESGCN achieves 87.84% accuracy, with 1.57% improvements over AGCN [24]. Figure 7 and Table 3 show that in all the 60 actions, there are 43 actions that achieve improvements by 9% to 1%, 8 actions have no changes, and only 9 actions get lower accuracies. We select two of these actions for in-depth analysis: “47.touch neck” and “44.touch head”. In AGCN [24], there are 77% of action “47.touch neck” samples are correctly classified, and 11% samples are misclassified as action “44.touch head”. In contrast, in our ESGCN, the first percentage rises to 84% and the second percentage drops to 3%. These two actions are illustrated in Figure 10. We can observe that they are all performed by raising one hand and touching the corresponding body part, which means that they are very similar in execution stages for the temporal domain. But in the spatial domain, these two actions can be easily classified by measuring the distance between the hand and the head or neck. Our spatial enhancement strategy can capture this difference more effectively.

To further demonstrate the effectiveness of our new block structure paradigm, we also propose another version of ESGCN named ESGCN V2 which is shown in Figure 6d. This version retains the traditional tandem structure while adding a spatial GCL in parallel. As shown in row 6 of Table 2, its accuracy is 87.52%, which is 1.25% higher than AGCN [24], proving the effectiveness of adding an additional spatial GCL. But it is 0.32% lower than ESGCN, so we adopt the latter structure in our final model.

#### 4.3.3. Enhanced Spatial and Extended Temporal Graph Convolution

In this section, we evaluate the performance of our final model. As shown in row 7 of Table 2, our EEGCN achieves 89.28% accuracy which is the highest in Table 2, with 3.01% improvements over AGCN [24]. The results also demonstrate the complementarity between our ESGCN and ETGCN. As shown in Figure 7 and Table 3, in all the 60 actions, up to 52 actions achieve improvements by 14% to 1%, four actions have no changes, and only four actions gave lower accuracies.

Here we also propose another version of EEGCN named EEGCN V2 which is shown in Figure 6e. It is constructed based on ETGCN, which adds an additional spatial GCL in parallel. As shown in row 8 of Table 2, its performance is 89.17%, which is 2.9% higher than AGCN [24], demonstrating the advantages of our enhanced spatial and extended temporal strategies. But it is 0.11% lower than EEGCN, so we adopt the latter version in our model.

To further evaluate the effectiveness of our EEGCN, we conduct it with all the four data modalities on both X-View and X-Sub benchmarks. The results are reported in Table 5. Comparing with AGCN [24], EEGCN brings significant improvements, ranging from 0.92% (joint motion) to 1.44% (bone) on X-View, and 2.78% (bone motion) to 3.01% (joint) on X-Sub. Since the accuracies on X-View are already very high, the improvements on X-Sub are relatively more significant.

### 4.4. Comparison to Other State-of-the-Art Methods

As introduced in Section 3.1, to make a fair comparison, we follow the traditional four-stream strategy which introduces four data modalities into our EEGCN. We denote our model using the joint data as 1s-EEGCN, our model using joint and bone data as 2s-EEGCN, our model using joint, bone, and their motions data as 4s-EEGCN. Note that although some methods [51,54] use only one stream, they fuse two types of information in the early stage to achieve better accuracies. To verify the superiority and generality of our method, we compare EEGCN with other state-of-the-art networks on NTU-RGB + D 60 [9], NTU-RGB + D 120 [38], and Kinetics-Skeleton [39]. We have also marked the number of streams used by these methods which are divided into two categories: non-GCN-based and GCN-based methods. The results are reported in Table 6, Table 7 and Table 8. 

We can observe that the GCN-based methods generally perform better than the traditional deep learning-based methods (i.e., CNNs and RNNs), demonstrating the effectiveness of incorporating the graph information into the network. For the NTU-RGB + D 60 dataset, our 1s-EEGCN achieves 89.3% accuracy on X-Sub, 95.3% accuracy on X-View, with 1.3% and 0.2% improvements respectively over 1s-AAGCN [28] which is also derived from AGCN [24]. Compared with all other methods, our 2s-EEGCN achieves the highest accuracy on X-Sub benchmark, even 0.4% higher over the current best performance method (i.e., 4s-Shift-GCN [30]). Our 4s-EEGCN improves the accuracies to a new level. For the NTU-RGB+D 120 dataset, our 1s-EEGCN is comparable with 2s-AGCN [24]. Our 2s-EEGCN exceeds all previously reported performance. Our 4s-EEGCN sets a new performance record again, which outperforms the current state-of-the-art 4s-Shift-GCN [30] at 1.5% on X-Sub, 1.3% on X-Set. Our method also shows its superiority on the Kinetics-Skeleton dataset which is more challenging. Overall, on all the three large-scale datasets, our three settings of EEGCN outperform other state-of-the-art methods using the same number of streams, especially our 4s-EEGCN is superior to all existing methods under all evaluation settings.

## 5. Conclusions

In this paper, we propose an enhanced spatial and extended temporal graph convolutional network for the skeleton-based action recognition task. For the temporal dimension, multiple relatively small kernel sizes are employed to extract temporal discriminative features. The corresponding layers are concatenated by a powerful yet efficient OSA + eSE structure. For the spatial dimension, a new paradigm of the block structure is proposed to enhance the spatial features, which expands the basic block structure from only tandem connection to a combination of tandem and parallel connections. Based on this, we add an additional spatial GCL in parallel with the temporal GCLs. Our method delivers state-of-the-art performance. In practical applications, our model can be applied to security surveillance systems, health care systems, and human-computer interaction systems, etc.

Despite the superiority of our EEGCN, there are still several issues to be addressed. Firstly, although our temporal extension strategy has been proved to be efficient, the efficiency of our spatial enhancement strategy still needs to be improved. Adding an additional spatial GCL will obviously increase the number of parameters of the model. It may be worth a try to reduce the number of spatial convolution channels or basic blocks. Secondly, the low-level features (i.e., joints, bones, and their motions) have shown their power in improving the performance of the model. However, the multi-stream construction is inefficient which doubles or even quadruples the number of parameters of the model. So we recommend more exploration of fusing the variable low-level features to just one stream. Thirdly, the joint semantics (e.g., frame index and joint type) can also provide discriminative information [54]. So the next step of our work will focus on how to incorporate the joint semantics into our EEGCN.

## Figures and Tables

**Table 1:** Action recognition accuracies by ETGCN which use multiple temporal convolution kernels with different sizes.

Kernel Sizes	Acc (%)	Params(M)
9 × 1	86.27	3.47
1 × 1, 9 × 1	88.34	2.66
3 × 1, 9 × 1	88.49	2.92
5 × 1, 9 × 1	88.58	3.19
1 × 1, 3 × 1, 9 × 1	88.56	2.99
1 × 1, 5 × 1, 9 × 1	88.68	3.12
3 × 1, 5 × 1, 9 × 1	88.33	3.38

**Table 2:** Action recognition accuracies by the various variants of our proposed method.

Methods	Acc (%)
Baseline (AGCN [24])	86.27
ETGCN	88.68(+2.41)
ETGCN V2	87.47(+1.20)
ESGCN	87.84(+1.57)
ESGCN V2	87.52(+1.25)
EEGCN	89.28(+3.01)
EEGCN V2	89.17(+2.90)

**Table 3:** The accuracy comparisons between ESGCN, ETGCN, EEGCN and AGCN [24]. Column 2/3/4 denotes how many classes of actions get better/the same/worse accuracies than AGCN [24].

Method	Better	The Same	Worse
ETGCN	47(78.3%)	4(6.7%)	9(15.0%)
ESGCN	43(71.7%)	8(13.3%)	9(15.0%)
EEGCN	52(86.7%)	4(6.7%)	4(6.7%)

**Table 4:** Confusion matrix for the action of “10.clapping” and “35.nod head/bow”.

Methods	Predicted Label Percentages (10.Clapping)						Predicted Label Percentages (35.Nod Head/Bow)					
	10	12	29	33	34	39	35	6	16	17	43	48
AGCN [24]	67%	3%	3%	1%	16%	2%	88%	3%	3%	2%	1%	1%
ETGCN	81%	0%	0%	0%	7%	1%	96%	0%	0%	0%	0%	0%

**Table 5:** The accuracy comparisons between AGCN [24] and EEGCN with four modalities on X-View and X-Sub benchmarks.

Methods	X-View (%)				X-Sub (%)			
	Joint	Bone	j-m	b-m	Joint	Bone	j-m	b-m
AGCN [24]	93.94	93.88	92.45	92.41	86.27	86.65	84.24	84.71
EEGCN	95.31 (+1.37)	95.32 (+1.44)	93.37 (+0.92)	93.62 (+1.21)	89.28 (+3.01)	89.46 (+2.81)	87.14 (+2.90)	87.49 (+2.78)

**Table 6:** The accuracy comparisons with state-of-the-art methods on the NUT-GRB + D 60 dataset.

Methods	Year	X-Sub (%)	X-View (%)
Ind-RNN [44]	2018	81.8	88.0
SR-TSL [10]	2018	84.8	92.4
HCN [51]	2018	86.5	91.1
ARRN-LSTM [43]	2019	81.8	89.6
AGC-LSTM [46]	2019	89.2	95.0
4s-MF-Net [52]	2019	90.0	95.4
AS-GCN [25]	2019	86.8	94.2
1s-AGCN [24]	2019	86.3	93.7
2s-AGCN [24]	2019	88.5	95.1
4s-DGNN [55]	2019	89.9	96.1
SGN [54]	2020	89.0	94.5
2s-GCN-NAS [33]	2020	89.4	95.7
1s-AAGCN [28]	2020	88.0	95.1
2s-AAGCN [28]	2020	89.4	96.0
4s-AAGCN [28]	2020	90.0	96.2
1s-Shift-GCN [30]	2020	87.8	95.1
2s-Shift-GCN [30]	2020	89.7	96.0
4s-Shift-GCN [30]	2020	90.7	96.5
1S-EEGCN (ours)	-	89.3	95.3
2S-EEGCN (ours)	-	91.1	96.4
4S-EEGCN (ours)	-	91.6	96.8

**Table 7:** The accuracy comparisons with state-of-the-art methods on the NUT-GRB + D 120 dataset.

Methods	Year	X-Sub (%)	X-Set (%)
ST-LSTM [19]	2016	55.7	57.9
GCA-LSTM [56]	2017	61.2	63.3
RotClips + MTCNN [57]	2018	62.2	61.8
Body Pose Evolution Map [58]	2018	64.6	66.9
2s-AGCN [24]	2019	82.9	84.9
SGN [54]	2020	79.2	81.5
1s-Shift-GCN [30]	2020	80.9	83.2
2s-Shift-GCN [30]	2020	85.3	86.6
4s-Shift-GCN [30]	2020	85.9	87.6
1S-EEGCN (ours)	-	82.5	84.2
2S-EEGCN (ours)	-	86.6	87.9
4S-EEGCN (ours)	-	87.4	88.9

**Table 8:** The accuracy comparisons with state-of-the-art methods on the Kinetics-Skeleton dataset.

Methods	Year	Top-1(%)	Top-5(%)
Deep LSTM [9]	2016	25.8	35.3
4s-MF-Net [52]	2019	33.2	55.5
AS-GCN [25]	2019	34.8	56.5
1s-AGCN [24]	2019	35.1	57.1
2s-AGCN [24]	2019	36.1	58.7
4s-DGNN [55]	2019	36.9	59.6
1s-GCN-NAS [33]	2020	35.5	57.9
2s-GCN-NAS [33]	2020	37.1	60.0
1s-AAGCN [28]	2020	36.0	58.4
2s-AAGCN [28]	2020	37.4	60.4
4s-AAGCN [28]	2020	37.8	61.0
1S-EEGCN (ours)	-	36.6	59.1
2S-EEGCN (ours)	-	38.3	60.0
4S-EEGCN (ours)	-	39.1	61.8
